# Absolute configuration of micromelin

**DOI:** 10.1107/S1600536811022720

**Published:** 2011-06-18

**Authors:** Hoong-Kun Fun, Ittipon Siridechakorn, Surat Laphookhieo, Suchada Chantrapromma

**Affiliations:** a X-ray Crystallography Unit, School of Physics, Universiti Sains Malaysia, 11800 USM, Penang, Malaysia; b Natural Products Research Laboratory, School of Science, Mae Fah Luang University, Tasud, Muang Chiang Rai 57100, Thailand; c Crystal Materials Research Unit, Department of Chemistry, Faculty of Science, Prince of Songkla University, Hat-Yai, Songkhla 90112, Thailand

## Abstract

The title compound {systematic name: 7-meth­oxy-6-[(1*R*,2*R*,5*R*)-5-methyl-4-oxo-3,6-dioxabicyclo­[3.1.0]hexan-2-yl]-2*H*-chromen-2-one}, C_15_H_12_O_6_, is a coumarin, which was isolated from the roots of *Micromelum glanduliferum*. There are two mol­ecules in the asymmetric unit with slight differences in bond angles. In both mol­ecules, the furan ring adopts a flattened envelope conformation. In the crystal, mol­ecules are linked by weak C—H⋯O inter­actions into chains along the *a* axis. Aromatic π–π stacking inter­actions with centroid–centroid distances in the range 3.6995 (11)–3.8069 (11) Å and C⋯O short contacts [3.030 (2)–3.171 (3) Å] also occur.

## Related literature

For bond-length data, see: Allen *et al.* (1987 ▶). For ring conformations, see: Cremer & Pople (1975 ▶). For background to plants in the Rutaceae family, coumarins and their activities, see: Ito *et al.* (1997 ▶, 2000 ▶); Kamperdick *et al.* (1999 ▶); Rahmani *et al.* (2003 ▶); Tangyuenyongwatthana *et al.* (1992 ▶); Tanti­shaiyakul *et al.* (1986 ▶); Tanti­vatana *et al.* (1983 ▶); Thuy *et al.* (1999 ▶). For the stability of the temperature controller used in the data collection, see Cosier & Glazer, (1986 ▶).
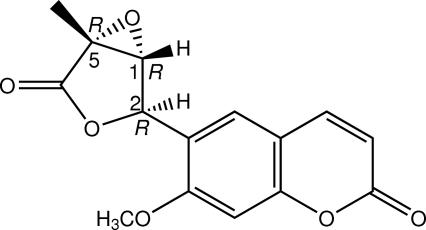


## Experimental

### 

#### Crystal data

C_15_H_12_O_6_
*M* *_r_* = 288.25
Monoclinic, 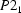

*a* = 6.7514 (2) Å
*b* = 23.7537 (8) Å
*c* = 8.0730 (3) Å
β = 90.000 (1)°
*V* = 1294.67 (8) Å^3^
*Z* = 4
Cu *K*α radiation
μ = 0.98 mm^−1^
*T* = 100 K
0.56 × 0.22 × 0.19 mm

#### Data collection

Bruker APEX DUO CCD diffractometer
Absorption correction: multi-scan (*SADABS*; Bruker, 2009 ▶) *T*_min_ = 0.609, *T*_max_ = 0.838
21511 measured reflections
4392 independent reflections
4392 reflections with *I* > 2σ(*I*)
*R*_int_ = 0.028

#### Refinement

*R*[*F* ^2^ > 2σ(*F* ^2^)] = 0.022
*wR*(*F* ^2^) = 0.059
*S* = 1.06
4392 reflections
384 parameters
1 restraint
H-atom parameters constrained
Δρ_max_ = 0.13 e Å^−3^
Δρ_min_ = −0.13 e Å^−3^
Absolute structure: Flack (1983 ▶), 2632 Friedel pairs
Flack parameter: 0.06 (10)

### 

Data collection: *APEX2* (Bruker, 2009 ▶); cell refinement: *SAINT* (Bruker, 2009 ▶); data reduction: *SAINT*; program(s) used to solve structure: *SHELXTL* (Sheldrick, 2008 ▶); program(s) used to refine structure: *SHELXTL*; molecular graphics: *SHELXTL*; software used to prepare material for publication: *SHELXTL* and *PLATON* (Spek, 2009 ▶).

## Supplementary Material

Crystal structure: contains datablock(s) global, I. DOI: 10.1107/S1600536811022720/hb5904sup1.cif

Structure factors: contains datablock(s) I. DOI: 10.1107/S1600536811022720/hb5904Isup2.hkl

Supplementary material file. DOI: 10.1107/S1600536811022720/hb5904Isup3.cml

Additional supplementary materials:  crystallographic information; 3D view; checkCIF report

## Figures and Tables

**Table 1:** Hydrogen-bond geometry (Å, °)

*D*—H⋯*A*	*D*—H	H⋯*A*	*D*⋯*A*	*D*—H⋯*A*
C3*B*—H3*B*⋯O4*A*^i^	0.93	2.60	3.450 (2)	152
C5*A*—H5*A*⋯O2*A*^ii^	0.93	2.60	3.518 (3)	171
C5*B*—H5*B*⋯O2*B*^iii^	0.93	2.57	3.493 (2)	173
C8*A*—H8*A*⋯O5*A*^iii^	0.93	2.58	3.440 (3)	155
C8*B*—H8*B*⋯O5*B*^ii^	0.93	2.42	3.298 (3)	157
C10*A*—H10*A*⋯O2*B*	0.98	2.35	3.186 (2)	142
C10*B*—H10*B*⋯O2*A*^iv^	0.98	2.29	3.171 (3)	150
C14*B*—H14*E*⋯O4*A*^v^	0.96	2.49	3.423 (3)	163
C15*B*—H15*D*⋯O4*A*^v^	0.96	2.46	3.405 (2)	166

Symmetry codes: (i) 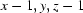; (ii) 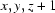; (iii) 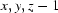; (iv) 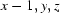; (v) 
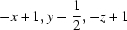
.

**Fig. 1.**
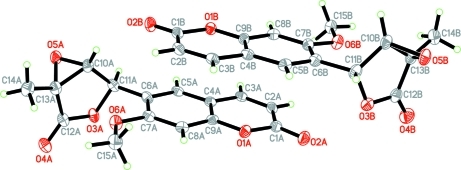
The structure of (I), showing 50% probability displacement ellipsoids.

**Fig. 2.**
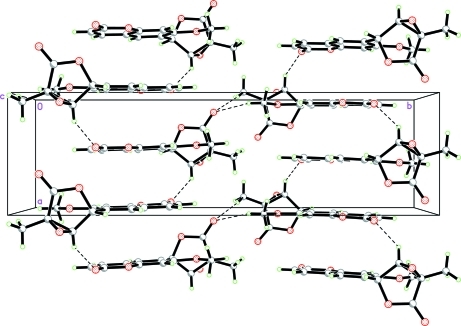
The crystal packing of (I) viewed along the c axis. Hydrogen bonds are shown as dashed lines.

## supplementary crystallographic information

### Comment

Coumarins are important secondary metabolites which occur commonly in the Rutaceae family. Many of them have been isolated from several genera of Rutaceae especially from *Micromelum* and *Clausena* genera (Ito *et al.*, 1997; 2000; Kamperdick *et al.*, 1999; Rahmani *et al.*, 2003; Tangyuenyongwatthana *et al.*, 1992; Tantishaiyakul *et al.*, 1986; Thuy *et al.*, 1999) and some of these compounds show interesting biological activities (Tantishaiyakul *et al.*, 1986; Tantivatana *et al.*, 1983). Although *Micromelum glanduliferum* is one of Rutaceae plants, however, no phytochemical investigation has been reported. As part of our continuing studies of the phytochemical constituents and bioactive compounds in Thai medicinal plants, we report herein the crystal structure of the title compound which was isolated from the roots of *M. glanduliferum* which were collected from Nongkai Province in the northeastern part of Thailand.

There are two crystallograpic independent molecules *A* and *B* in the asymmetric unit of (I), C_15_H_12_O_6_, (Fig. 1) with the same conformation but with slight differences in bond angles. In the structure of (I), the furan ring (C10–C13/O3) is in a flattened envelope conformation with the puckering atom O3 of 0.064 (1) Å, and puckering parameter Q = 0.0991 (18) Å and φ = 9.2 (11)° (Cremer & Pople, 1975) for molecule *A* and the corresponding values are -0.053 (1) Å, 0.0820 (19) Å and φ = 10.1 (15)° for molecule *B*. The benzene and dihydro-pyran ring system (C1–C9/O1) is planar with the *r.m.s*. 0.0089 (2) Å for molecule *A* [0.0149 (2) Å for molecule *B*]. The methoxy group is almost planarly attached to the benzene ring with the torsion angle C15–O6–C7–C8 = 2.3 (3)° for molecule *A* and 2.6 (3)° for molecule *B*. The orientation of the oxirane ring (C10–C13–O5) can be indicated by the dihedral angle between the furan and oxirane rings being 79.46 (15)° for molecule *A* [79.48 (16)° for molecule *B*]. The bond distances in (I) are within normal ranges (Allen *et al.*, 1987). The absolute configuration at atoms C10, C11 and C13 or positions 1, 2 and 5 of the micromelin are *R*,*R*,*R* configurations.

In the crystal (Fig. 2), molecules are linked by C—H···O weak interactions into 2D chains along the *a* axis. π–π interactions were observed with centroid···centroid distances: Cg_1_···Cg_3_ = 3.7698 (7) Å; Cg_2_···Cg_5_ = 3.7102 (11) Å; Cg_3_···Cg_4_ = 3.6995 (11) Å and 3.7666 (11) Å (symmetry code: 1+x, y, z); Cg_1_, Cg_2_, Cg_3_, Cg_4_ and Cg_5_ are the centroids of C10A–C13A/O3A, C1A–C4A/C9A/O1A, C4A–C9A, C1B–C4B/C9B/O1B and C4B–C9B rings, respectively. C···O [3.030 (2)-3.171 (3) Å] short contacts were also observed.

### Experimental

The chemical contents of the roots of *M. glanduliferum* (5.25 kg) were successively extracted with CH_2_Cl_2_ over the period of 3 days at room temperature. Removal the solvent under reduced pressure provided CH_2_Cl_2_ extract which were subjected to quick column chromatography (QCC) over silica gel and eluted with a gradient of n-hexane-EtOAc (100% n-hexane to 100% EtOAc) to provide nine fractions (A-I). Fraction F (6.23 g) was washed with n-hexane and recrystallized from CH_2_Cl_2_/CH_3_OH (1:4 v/v) to give colourless needles of the title compound. Mp 491.0-492.2 K (decompose).

### Refinement

H atoms were placed in calculated positions with (C—H) = 0.93 for aromatic and 0.96 Å for CH_3_ atoms. The *U*_iso_ values were constrained to be 1.5*U*_eq_ of the carrier atom for methyl H atoms and 1.2*U*_eq_ for the remaining H atoms. A rotating group model was used for the methyl groups. The highest residual electron density peak is located at 0.79 Å from C5B and the deepest hole is located at 0.81 Å from C7B. 2632 Friedel pairs were used to determine the absolute configuration. The crystal is a twin with BASF = 0.497 (1).

### Crystal data

**Table:**

C_15_H_12_O_6_	*F*(000) = 600
*M**_r_* = 288.25	*D*_x_ = 1.479 Mg m^−^^3^
Monoclinic, *P*2_1_	Melting point = 491.0–492.2 (decompose) K
Hall symbol: P 2yb	Cu *K*α radiation, λ = 1.54178 Å
*a* = 6.7514 (2) Å	Cell parameters from 4392 reflections
*b* = 23.7537 (8) Å	θ = 1.9–67.5°
*c* = 8.0730 (3) Å	µ = 0.98 mm^−^^1^
β = 90.000 (1)°	*T* = 100 K
*V* = 1294.67 (8) Å^3^	Needle, colorless
*Z* = 4	0.56 × 0.22 × 0.19 mm

### Data collection

**Table:**

Bruker APEX DUO CCD diffractometer	4392 independent reflections
Radiation source: sealed tube	4392 reflections with *I* > 2σ(*I*)
graphite	*R*_int_ = 0.028
φ and ω scans	θ_max_ = 67.5°, θ_min_ = 1.9°
Absorption correction: multi-scan (*SADABS*; Bruker, 2009)	*h* = −8→8
*T*_min_ = 0.609, *T*_max_ = 0.838	*k* = −28→28
21511 measured reflections	*l* = −7→9

### Refinement

**Table:**

Refinement on *F*^2^	Secondary atom site location: difference Fourier map
Least-squares matrix: full	Hydrogen site location: inferred from neighbouring sites
*R*[*F*^2^ > 2σ(*F*^2^)] = 0.022	H-atom parameters constrained
*wR*(*F*^2^) = 0.059	
*S* = 1.06	(Δ/σ)_max_ = 0.001
4392 reflections	Δρ_max_ = 0.13 e Å^−^^3^
384 parameters	Δρ_min_ = −0.13 e Å^−^^3^
1 restraint	Absolute structure: Flack (1983), 2632 Friedel pairs
Primary atom site location: structure-invariant direct methods	Flack parameter: 0.06 (10)

### Special details

**Table:**

Experimental. The crystal was placed in the cold stream of an Oxford Cryosystems Cobra open-flow nitrogen cryostat (Cosier & Glazer, 1986) operating at 100.0 (1) K.
Geometry. All esds (except the esd in the dihedral angle between two l.s. planes) are estimated using the full covariance matrix. The cell esds are taken into account individually in the estimation of esds in distances, angles and torsion angles; correlations between esds in cell parameters are only used when they are defined by crystal symmetry. An approximate (isotropic) treatment of cell esds is used for estimating esds involving l.s. planes.
Refinement. Refinement of F^2^ against ALL reflections. The weighted R-factor wR and goodness of fit S are based on F^2^, conventional R-factors R are based on F, with F set to zero for negative F^2^. The threshold expression of F^2^ > 2sigma(F^2^) is used only for calculating R-factors(gt) etc. and is not relevant to the choice of reflections for refinement. R-factors based on F^2^ are statistically about twice as large as those based on F, and R- factors based on ALL data will be even larger.

### Fractional atomic coordinates and isotropic or equivalent isotropic displacement parameters (Å^2^)

**Table:**

	*x*	*y*	*z*	*U*_iso_*/*U*_eq_	
O1A	0.5534 (2)	0.77310 (5)	0.20837 (17)	0.0204 (3)	
O2A	0.5534 (2)	0.70240 (6)	0.03012 (18)	0.0264 (3)	
O3A	0.74087 (18)	0.88672 (5)	0.89684 (16)	0.0216 (3)	
O4A	0.8825 (2)	0.97077 (5)	0.92773 (18)	0.0269 (3)	
O5A	0.4227 (2)	0.93361 (5)	1.05332 (16)	0.0241 (3)	
O6A	0.5758 (2)	0.93052 (5)	0.57220 (16)	0.0241 (3)	
C1A	0.5487 (3)	0.71597 (7)	0.1741 (2)	0.0202 (4)	
C2A	0.5373 (2)	0.67836 (8)	0.3155 (3)	0.0214 (4)	
H2A	0.5333	0.6397	0.2974	0.026*	
C3A	0.5323 (3)	0.69786 (8)	0.4722 (2)	0.0201 (4)	
H3A	0.5235	0.6727	0.5602	0.024*	
C4A	0.5405 (3)	0.75717 (7)	0.5038 (2)	0.0181 (4)	
C5A	0.5383 (3)	0.78164 (8)	0.6622 (3)	0.0181 (4)	
H5A	0.5292	0.7584	0.7546	0.022*	
C6A	0.5493 (3)	0.83936 (7)	0.6845 (2)	0.0195 (4)	
C7A	0.5631 (3)	0.87404 (7)	0.5419 (2)	0.0189 (4)	
C8A	0.5625 (3)	0.85146 (8)	0.3842 (3)	0.0195 (4)	
H8A	0.5690	0.8746	0.2914	0.023*	
C9A	0.5519 (3)	0.79334 (8)	0.3677 (2)	0.0182 (4)	
C10A	0.4044 (3)	0.91242 (8)	0.8848 (2)	0.0211 (4)	
H10A	0.2787	0.9151	0.8246	0.025*	
C11A	0.5444 (3)	0.86343 (7)	0.8562 (2)	0.0215 (4)	
H11A	0.5128	0.8333	0.9349	0.026*	
C12A	0.7343 (3)	0.94293 (7)	0.9141 (2)	0.0212 (3)	
C13A	0.5240 (3)	0.96269 (8)	0.9180 (2)	0.0225 (4)	
C14A	0.4710 (3)	1.02263 (7)	0.8862 (2)	0.0281 (4)	
H14A	0.3296	1.0268	0.8891	0.042*	
H14B	0.5195	1.0337	0.7793	0.042*	
H14C	0.5297	1.0460	0.9698	0.042*	
C15A	0.5814 (3)	0.96784 (8)	0.4322 (2)	0.0272 (4)	
H15A	0.5919	1.0060	0.4703	0.041*	
H15B	0.4623	0.9635	0.3686	0.041*	
H15C	0.6938	0.9589	0.3643	0.041*	
O1B	0.05200 (18)	0.79463 (5)	0.46409 (16)	0.0183 (3)	
O2B	0.0717 (2)	0.86476 (5)	0.64252 (16)	0.0242 (3)	
O3B	0.23060 (19)	0.67681 (5)	−0.22314 (17)	0.0233 (3)	
O4B	0.34079 (19)	0.58955 (6)	−0.2685 (2)	0.0318 (3)	
O5B	−0.09923 (19)	0.64242 (5)	−0.38897 (15)	0.0236 (3)	
O6B	0.04861 (19)	0.63788 (5)	0.09768 (17)	0.0225 (3)	
C1B	0.0630 (3)	0.85163 (7)	0.4988 (2)	0.0203 (4)	
C2B	0.0651 (3)	0.88964 (7)	0.3574 (2)	0.0199 (4)	
H2B	0.0720	0.9282	0.3761	0.024*	
C3B	0.0575 (3)	0.87071 (7)	0.2008 (2)	0.0197 (4)	
H3B	0.0557	0.8961	0.1131	0.024*	
C4B	0.0519 (3)	0.81105 (8)	0.1684 (2)	0.0178 (4)	
C5B	0.0489 (3)	0.78703 (8)	0.0093 (2)	0.0199 (4)	
H5B	0.0482	0.8104	−0.0831	0.024*	
C6B	0.0471 (3)	0.72951 (8)	−0.0123 (2)	0.0194 (4)	
C7B	0.0466 (3)	0.69415 (7)	0.1295 (2)	0.0192 (4)	
C8B	0.0462 (3)	0.71642 (7)	0.2875 (3)	0.0175 (3)	
H8B	0.0440	0.6931	0.3801	0.021*	
C9B	0.0491 (3)	0.77488 (7)	0.3041 (2)	0.0179 (4)	
C10B	−0.1127 (3)	0.66169 (7)	−0.2188 (3)	0.0215 (4)	
H10B	−0.2398	0.6622	−0.1602	0.026*	
C11B	0.0431 (3)	0.70558 (9)	−0.1849 (2)	0.0228 (4)	
H11B	0.0254	0.7366	−0.2636	0.027*	
C12B	0.2048 (3)	0.62152 (8)	−0.2490 (2)	0.0226 (4)	
C13B	−0.0122 (3)	0.60858 (8)	−0.2562 (2)	0.0207 (4)	
C14B	−0.0890 (3)	0.55004 (8)	−0.2368 (3)	0.0278 (4)	
H14D	−0.2311	0.5507	−0.2342	0.042*	
H14E	−0.0399	0.5343	−0.1353	0.042*	
H14F	−0.0453	0.5275	−0.3284	0.042*	
C15B	0.0417 (3)	0.60079 (7)	0.2381 (2)	0.0223 (4)	
H15D	0.0417	0.5624	0.2007	0.033*	
H15E	−0.0765	0.6079	0.3007	0.033*	
H15F	0.1555	0.6072	0.3069	0.033*	

### Atomic displacement parameters (Å^2^)

**Table:**

	*U*^11^	*U*^22^	*U*^33^	*U*^12^	*U*^13^	*U*^23^
O1A	0.0222 (6)	0.0233 (7)	0.0158 (7)	0.0021 (5)	−0.0004 (5)	−0.0002 (5)
O2A	0.0267 (7)	0.0324 (7)	0.0201 (8)	0.0025 (6)	−0.0010 (6)	−0.0059 (6)
O3A	0.0261 (6)	0.0169 (5)	0.0217 (7)	−0.0002 (5)	−0.0085 (6)	−0.0002 (5)
O4A	0.0280 (7)	0.0249 (6)	0.0278 (8)	−0.0054 (5)	−0.0071 (6)	−0.0003 (5)
O5A	0.0309 (7)	0.0241 (6)	0.0171 (6)	−0.0045 (5)	0.0002 (5)	−0.0034 (5)
O6A	0.0348 (7)	0.0188 (6)	0.0188 (7)	−0.0023 (5)	−0.0023 (6)	0.0039 (5)
C1A	0.0148 (8)	0.0238 (9)	0.0219 (11)	0.0021 (7)	−0.0029 (7)	−0.0024 (7)
C2A	0.0169 (8)	0.0224 (8)	0.0248 (10)	−0.0003 (7)	−0.0031 (7)	−0.0036 (7)
C3A	0.0148 (8)	0.0233 (9)	0.0223 (11)	−0.0016 (7)	−0.0022 (7)	0.0022 (7)
C4A	0.0138 (8)	0.0209 (9)	0.0197 (9)	−0.0004 (6)	−0.0014 (7)	0.0021 (7)
C5A	0.0149 (8)	0.0194 (8)	0.0201 (9)	−0.0013 (6)	−0.0030 (7)	0.0050 (6)
C6A	0.0174 (8)	0.0210 (9)	0.0202 (10)	−0.0014 (7)	−0.0064 (7)	0.0011 (7)
C7A	0.0172 (8)	0.0193 (9)	0.0202 (10)	−0.0008 (7)	−0.0029 (7)	0.0035 (7)
C8A	0.0173 (8)	0.0232 (9)	0.0179 (9)	0.0001 (7)	−0.0019 (7)	0.0045 (7)
C9A	0.0126 (8)	0.0243 (9)	0.0178 (10)	0.0007 (7)	−0.0013 (8)	0.0004 (7)
C10A	0.0265 (8)	0.0225 (8)	0.0142 (9)	−0.0010 (8)	−0.0023 (7)	−0.0007 (7)
C11A	0.0248 (8)	0.0204 (9)	0.0193 (10)	−0.0049 (7)	−0.0051 (8)	0.0039 (7)
C12A	0.0297 (9)	0.0212 (8)	0.0127 (9)	−0.0039 (7)	−0.0063 (7)	0.0037 (6)
C13A	0.0298 (9)	0.0233 (8)	0.0143 (9)	−0.0020 (8)	−0.0036 (7)	0.0011 (7)
C14A	0.0325 (10)	0.0261 (10)	0.0258 (10)	0.0044 (8)	0.0000 (8)	−0.0006 (7)
C15A	0.0388 (11)	0.0206 (8)	0.0222 (10)	−0.0016 (8)	0.0005 (8)	0.0067 (7)
O1B	0.0193 (6)	0.0204 (6)	0.0153 (7)	0.0001 (5)	−0.0028 (5)	−0.0005 (5)
O2B	0.0288 (7)	0.0248 (7)	0.0192 (7)	−0.0029 (6)	−0.0037 (5)	−0.0047 (5)
O3B	0.0241 (6)	0.0253 (6)	0.0205 (7)	−0.0007 (5)	0.0035 (5)	−0.0015 (5)
O4B	0.0288 (7)	0.0317 (7)	0.0349 (8)	0.0085 (6)	0.0011 (6)	−0.0057 (6)
O5B	0.0318 (7)	0.0250 (6)	0.0139 (6)	0.0045 (5)	−0.0027 (5)	−0.0007 (5)
O6B	0.0306 (7)	0.0187 (6)	0.0181 (7)	0.0036 (5)	−0.0002 (6)	0.0005 (5)
C1B	0.0145 (8)	0.0204 (8)	0.0261 (12)	−0.0001 (7)	−0.0020 (7)	−0.0038 (7)
C2B	0.0171 (8)	0.0168 (8)	0.0259 (10)	−0.0012 (7)	−0.0040 (7)	−0.0007 (7)
C3B	0.0154 (8)	0.0204 (8)	0.0232 (10)	−0.0007 (7)	−0.0019 (8)	0.0027 (7)
C4B	0.0141 (8)	0.0191 (9)	0.0202 (10)	0.0007 (6)	−0.0027 (7)	0.0029 (7)
C5B	0.0186 (8)	0.0239 (9)	0.0172 (9)	0.0020 (7)	−0.0001 (7)	0.0037 (7)
C6B	0.0184 (8)	0.0212 (9)	0.0185 (10)	0.0031 (7)	−0.0023 (8)	0.0003 (7)
C7B	0.0166 (8)	0.0178 (8)	0.0231 (10)	0.0028 (7)	−0.0018 (7)	−0.0016 (7)
C8B	0.0163 (7)	0.0177 (8)	0.0184 (9)	0.0028 (6)	−0.0018 (7)	0.0054 (6)
C9B	0.0157 (8)	0.0221 (9)	0.0161 (9)	0.0005 (7)	−0.0021 (7)	−0.0026 (7)
C10B	0.0253 (9)	0.0258 (9)	0.0133 (9)	0.0044 (7)	−0.0019 (7)	−0.0013 (6)
C11B	0.0284 (9)	0.0226 (9)	0.0173 (10)	0.0050 (8)	0.0007 (8)	0.0007 (7)
C12B	0.0295 (9)	0.0239 (9)	0.0145 (10)	0.0013 (7)	0.0005 (7)	0.0008 (7)
C13B	0.0255 (8)	0.0238 (9)	0.0127 (10)	0.0033 (7)	−0.0010 (7)	0.0009 (7)
C14B	0.0339 (10)	0.0252 (9)	0.0243 (10)	−0.0033 (8)	−0.0034 (9)	−0.0007 (7)
C15B	0.0284 (8)	0.0188 (8)	0.0197 (9)	0.0032 (7)	−0.0017 (8)	0.0021 (7)

### Geometric parameters (Å, °)

**Table:**

O1A—C9A	1.373 (2)	O1B—C9B	1.374 (2)
O1A—C1A	1.385 (2)	O1B—C1B	1.385 (2)
O2A—C1A	1.207 (2)	O2B—C1B	1.203 (2)
O3A—C12A	1.343 (2)	O3B—C12B	1.341 (2)
O3A—C11A	1.474 (2)	O3B—C11B	1.471 (2)
O4A—C12A	1.204 (2)	O4B—C12B	1.202 (2)
O5A—C10A	1.456 (2)	O5B—C10B	1.451 (2)
O5A—C13A	1.462 (2)	O5B—C13B	1.463 (2)
O6A—C7A	1.366 (2)	O6B—C7B	1.361 (2)
O6A—C15A	1.437 (2)	O6B—C15B	1.437 (2)
C1A—C2A	1.451 (3)	C1B—C2B	1.455 (3)
C2A—C3A	1.348 (3)	C2B—C3B	1.343 (3)
C2A—H2A	0.9300	C2B—H2B	0.9300
C3A—C4A	1.433 (2)	C3B—C4B	1.441 (2)
C3A—H3A	0.9300	C3B—H3B	0.9300
C4A—C9A	1.397 (3)	C4B—C9B	1.392 (2)
C4A—C5A	1.405 (3)	C4B—C5B	1.406 (3)
C5A—C6A	1.385 (2)	C5B—C6B	1.377 (3)
C5A—H5A	0.9300	C5B—H5B	0.9300
C6A—C7A	1.419 (2)	C6B—C7B	1.420 (3)
C6A—C11A	1.500 (3)	C6B—C11B	1.505 (3)
C7A—C8A	1.381 (3)	C7B—C8B	1.381 (3)
C8A—C9A	1.389 (3)	C8B—C9B	1.395 (2)
C8A—H8A	0.9300	C8B—H8B	0.9300
C10A—C13A	1.466 (3)	C10B—C13B	1.464 (3)
C10A—C11A	1.517 (3)	C10B—C11B	1.506 (3)
C10A—H10A	0.9800	C10B—H10B	0.9800
C11A—H11A	0.9800	C11B—H11B	0.9800
C12A—C13A	1.496 (3)	C12B—C13B	1.498 (2)
C13A—C14A	1.490 (2)	C13B—C14B	1.492 (2)
C14A—H14A	0.9600	C14B—H14D	0.9600
C14A—H14B	0.9600	C14B—H14E	0.9600
C14A—H14C	0.9600	C14B—H14F	0.9600
C15A—H15A	0.9600	C15B—H15D	0.9600
C15A—H15B	0.9600	C15B—H15E	0.9600
C15A—H15C	0.9600	C15B—H15F	0.9600
			
C9A—O1A—C1A	121.99 (14)	C9B—O1B—C1B	121.63 (13)
C12A—O3A—C11A	111.50 (14)	C12B—O3B—C11B	112.06 (15)
C10A—O5A—C13A	60.32 (11)	C10B—O5B—C13B	60.31 (11)
C7A—O6A—C15A	117.82 (14)	C7B—O6B—C15B	116.94 (15)
O2A—C1A—O1A	116.95 (16)	O2B—C1B—O1B	116.82 (16)
O2A—C1A—C2A	126.46 (16)	O2B—C1B—C2B	126.52 (16)
O1A—C1A—C2A	116.58 (16)	O1B—C1B—C2B	116.66 (16)
C3A—C2A—C1A	121.86 (17)	C3B—C2B—C1B	122.02 (16)
C3A—C2A—H2A	119.1	C3B—C2B—H2B	119.0
C1A—C2A—H2A	119.1	C1B—C2B—H2B	119.0
C2A—C3A—C4A	120.27 (17)	C2B—C3B—C4B	120.06 (16)
C2A—C3A—H3A	119.9	C2B—C3B—H3B	120.0
C4A—C3A—H3A	119.9	C4B—C3B—H3B	120.0
C9A—C4A—C5A	117.51 (16)	C9B—C4B—C5B	117.92 (16)
C9A—C4A—C3A	117.85 (18)	C9B—C4B—C3B	117.67 (17)
C5A—C4A—C3A	124.65 (16)	C5B—C4B—C3B	124.41 (16)
C6A—C5A—C4A	121.84 (18)	C6B—C5B—C4B	121.22 (17)
C6A—C5A—H5A	119.1	C6B—C5B—H5B	119.4
C4A—C5A—H5A	119.1	C4B—C5B—H5B	119.4
C5A—C6A—C7A	118.23 (17)	C5B—C6B—C7B	119.00 (17)
C5A—C6A—C11A	119.76 (17)	C5B—C6B—C11B	119.47 (17)
C7A—C6A—C11A	122.01 (15)	C7B—C6B—C11B	121.53 (16)
O6A—C7A—C8A	123.13 (16)	O6B—C7B—C8B	123.40 (16)
O6A—C7A—C6A	115.41 (15)	O6B—C7B—C6B	115.39 (16)
C8A—C7A—C6A	121.47 (16)	C8B—C7B—C6B	121.20 (16)
C7A—C8A—C9A	118.33 (16)	C7B—C8B—C9B	118.02 (16)
C7A—C8A—H8A	120.8	C7B—C8B—H8B	121.0
C9A—C8A—H8A	120.8	C9B—C8B—H8B	121.0
O1A—C9A—C8A	115.95 (15)	O1B—C9B—C4B	121.91 (16)
O1A—C9A—C4A	121.44 (16)	O1B—C9B—C8B	115.46 (15)
C8A—C9A—C4A	122.62 (17)	C4B—C9B—C8B	122.62 (17)
O5A—C10A—C13A	60.06 (12)	O5B—C10B—C13B	60.26 (12)
O5A—C10A—C11A	110.76 (14)	O5B—C10B—C11B	110.29 (15)
C13A—C10A—C11A	108.03 (15)	C13B—C10B—C11B	108.09 (15)
O5A—C10A—H10A	120.9	O5B—C10B—H10B	121.0
C13A—C10A—H10A	120.9	C13B—C10B—H10B	121.0
C11A—C10A—H10A	120.9	C11B—C10B—H10B	121.0
O3A—C11A—C6A	109.19 (15)	O3B—C11B—C6B	110.73 (15)
O3A—C11A—C10A	103.82 (14)	O3B—C11B—C10B	103.96 (15)
C6A—C11A—C10A	116.54 (15)	C6B—C11B—C10B	116.25 (17)
O3A—C11A—H11A	109.0	O3B—C11B—H11B	108.5
C6A—C11A—H11A	109.0	C6B—C11B—H11B	108.5
C10A—C11A—H11A	109.0	C10B—C11B—H11B	108.5
O4A—C12A—O3A	121.87 (16)	O4B—C12B—O3B	122.68 (18)
O4A—C12A—C13A	127.90 (16)	O4B—C12B—C13B	127.76 (17)
O3A—C12A—C13A	110.21 (15)	O3B—C12B—C13B	109.51 (16)
O5A—C13A—C10A	59.62 (11)	O5B—C13B—C10B	59.43 (11)
O5A—C13A—C14A	117.86 (16)	O5B—C13B—C14B	116.71 (15)
C10A—C13A—C14A	127.90 (17)	C10B—C13B—C14B	128.36 (16)
O5A—C13A—C12A	108.14 (14)	O5B—C13B—C12B	107.97 (15)
C10A—C13A—C12A	105.27 (15)	C10B—C13B—C12B	105.57 (15)
C14A—C13A—C12A	121.63 (16)	C14B—C13B—C12B	121.78 (15)
C13A—C14A—H14A	109.5	C13B—C14B—H14D	109.5
C13A—C14A—H14B	109.5	C13B—C14B—H14E	109.5
H14A—C14A—H14B	109.5	H14D—C14B—H14E	109.5
C13A—C14A—H14C	109.5	C13B—C14B—H14F	109.5
H14A—C14A—H14C	109.5	H14D—C14B—H14F	109.5
H14B—C14A—H14C	109.5	H14E—C14B—H14F	109.5
O6A—C15A—H15A	109.5	O6B—C15B—H15D	109.5
O6A—C15A—H15B	109.5	O6B—C15B—H15E	109.5
H15A—C15A—H15B	109.5	H15D—C15B—H15E	109.5
O6A—C15A—H15C	109.5	O6B—C15B—H15F	109.5
H15A—C15A—H15C	109.5	H15D—C15B—H15F	109.5
H15B—C15A—H15C	109.5	H15E—C15B—H15F	109.5
			
C9A—O1A—C1A—O2A	−178.97 (17)	C9B—O1B—C1B—O2B	−177.49 (14)
C9A—O1A—C1A—C2A	1.4 (3)	C9B—O1B—C1B—C2B	1.9 (2)
O2A—C1A—C2A—C3A	−179.84 (19)	O2B—C1B—C2B—C3B	179.08 (18)
O1A—C1A—C2A—C3A	−0.3 (3)	O1B—C1B—C2B—C3B	−0.2 (2)
C1A—C2A—C3A—C4A	−0.7 (3)	C1B—C2B—C3B—C4B	−1.6 (3)
C2A—C3A—C4A—C9A	0.6 (3)	C2B—C3B—C4B—C9B	1.8 (3)
C2A—C3A—C4A—C5A	−179.32 (16)	C2B—C3B—C4B—C5B	−178.27 (16)
C9A—C4A—C5A—C6A	−0.8 (3)	C9B—C4B—C5B—C6B	−1.2 (3)
C3A—C4A—C5A—C6A	179.12 (16)	C3B—C4B—C5B—C6B	178.86 (17)
C4A—C5A—C6A—C7A	0.0 (3)	C4B—C5B—C6B—C7B	0.5 (3)
C4A—C5A—C6A—C11A	179.40 (16)	C4B—C5B—C6B—C11B	179.74 (17)
C15A—O6A—C7A—C8A	2.3 (3)	C15B—O6B—C7B—C8B	2.6 (3)
C15A—O6A—C7A—C6A	−177.34 (16)	C15B—O6B—C7B—C6B	−178.06 (14)
C5A—C6A—C7A—O6A	−179.27 (15)	C5B—C6B—C7B—O6B	−178.79 (15)
C11A—C6A—C7A—O6A	1.3 (3)	C11B—C6B—C7B—O6B	2.0 (3)
C5A—C6A—C7A—C8A	1.1 (3)	C5B—C6B—C7B—C8B	0.6 (3)
C11A—C6A—C7A—C8A	−178.34 (17)	C11B—C6B—C7B—C8B	−178.65 (17)
O6A—C7A—C8A—C9A	179.09 (16)	O6B—C7B—C8B—C9B	178.41 (14)
C6A—C7A—C8A—C9A	−1.3 (3)	C6B—C7B—C8B—C9B	−0.9 (3)
C1A—O1A—C9A—C8A	178.32 (14)	C1B—O1B—C9B—C4B	−1.7 (3)
C1A—O1A—C9A—C4A	−1.6 (3)	C1B—O1B—C9B—C8B	177.39 (14)
C7A—C8A—C9A—O1A	−179.47 (16)	C5B—C4B—C9B—O1B	179.89 (14)
C7A—C8A—C9A—C4A	0.4 (3)	C3B—C4B—C9B—O1B	−0.1 (3)
C5A—C4A—C9A—O1A	−179.54 (16)	C5B—C4B—C9B—C8B	0.8 (3)
C3A—C4A—C9A—O1A	0.6 (3)	C3B—C4B—C9B—C8B	−179.19 (14)
C5A—C4A—C9A—C8A	0.6 (3)	C7B—C8B—C9B—O1B	−178.93 (14)
C3A—C4A—C9A—C8A	−179.34 (16)	C7B—C8B—C9B—C4B	0.2 (3)
C13A—O5A—C10A—C11A	99.42 (16)	C13B—O5B—C10B—C11B	99.79 (16)
C12A—O3A—C11A—C6A	113.83 (16)	C12B—O3B—C11B—C6B	116.25 (18)
C12A—O3A—C11A—C10A	−11.11 (18)	C12B—O3B—C11B—C10B	−9.3 (2)
C5A—C6A—C11A—O3A	110.37 (17)	C5B—C6B—C11B—O3B	112.02 (18)
C7A—C6A—C11A—O3A	−70.2 (2)	C7B—C6B—C11B—O3B	−68.8 (2)
C5A—C6A—C11A—C10A	−132.47 (18)	C5B—C6B—C11B—C10B	−129.66 (19)
C7A—C6A—C11A—C10A	46.9 (2)	C7B—C6B—C11B—C10B	49.6 (2)
O5A—C10A—C11A—O3A	−56.68 (17)	O5B—C10B—C11B—O3B	−57.98 (18)
C13A—C10A—C11A—O3A	7.34 (19)	C13B—C10B—C11B—O3B	6.2 (2)
O5A—C10A—C11A—C6A	−176.76 (14)	O5B—C10B—C11B—C6B	−179.94 (14)
C13A—C10A—C11A—C6A	−112.74 (17)	C13B—C10B—C11B—C6B	−115.77 (19)
C11A—O3A—C12A—O4A	−170.99 (17)	C11B—O3B—C12B—O4B	−173.74 (18)
C11A—O3A—C12A—C13A	10.6 (2)	C11B—O3B—C12B—C13B	8.7 (2)
C10A—O5A—C13A—C14A	119.70 (18)	C10B—O5B—C13B—C14B	120.65 (18)
C10A—O5A—C13A—C12A	−97.43 (17)	C10B—O5B—C13B—C12B	−97.83 (16)
C11A—C10A—C13A—O5A	−104.05 (15)	C11B—C10B—C13B—O5B	−103.51 (16)
O5A—C10A—C13A—C14A	−103.3 (2)	O5B—C10B—C13B—C14B	−101.4 (2)
C11A—C10A—C13A—C14A	152.64 (19)	C11B—C10B—C13B—C14B	155.06 (19)
O5A—C10A—C13A—C12A	102.35 (16)	O5B—C10B—C13B—C12B	101.97 (16)
C11A—C10A—C13A—C12A	−1.7 (2)	C11B—C10B—C13B—C12B	−1.5 (2)
O4A—C12A—C13A—O5A	−121.19 (19)	O4B—C12B—C13B—O5B	−119.4 (2)
O3A—C12A—C13A—O5A	57.12 (19)	O3B—C12B—C13B—O5B	58.02 (19)
O4A—C12A—C13A—C10A	176.34 (19)	O4B—C12B—C13B—C10B	178.3 (2)
O3A—C12A—C13A—C10A	−5.3 (2)	O3B—C12B—C13B—C10B	−4.3 (2)
O4A—C12A—C13A—C14A	20.0 (3)	O4B—C12B—C13B—C14B	19.8 (3)
O3A—C12A—C13A—C14A	−161.68 (15)	O3B—C12B—C13B—C14B	−162.80 (16)

### Hydrogen-bond geometry (Å, °)

**Table:**

*D*—H···*A*	*D*—H	H···*A*	*D*···*A*	*D*—H···*A*
C3B—H3B···O4A^i^	0.93	2.60	3.450 (2)	152
C5A—H5A···O2A^ii^	0.93	2.60	3.518 (3)	171
C5B—H5B···O2B^iii^	0.93	2.57	3.493 (2)	173
C8A—H8A···O5A^iii^	0.93	2.58	3.440 (3)	155
C8B—H8B···O5B^ii^	0.93	2.42	3.298 (3)	157
C10A—H10A···O2B	0.98	2.35	3.186 (2)	142
C10B—H10B···O2A^iv^	0.98	2.29	3.171 (3)	150
C14B—H14E···O4A^v^	0.96	2.49	3.423 (3)	163
C15B—H15D···O4A^v^	0.96	2.46	3.405 (2)	166

Symmetry codes: (i) *x*−1, *y*, *z*−1; (ii) *x*, *y*, *z*+1; (iii) *x*, *y*, *z*−1;  (iv) *x*−1, *y*, *z*; (v) −*x*+1, *y*−1/2, −*z*+1.
